# Innate Immunity Induces the Accumulation of Lung Mast Cells During Influenza Infection

**DOI:** 10.3389/fimmu.2018.02288

**Published:** 2018-10-04

**Authors:** Behdad Zarnegar, Annika Westin, Syrmoula Evangelidou, Jenny Hallgren

**Affiliations:** Department of Medical Biochemistry and Microbiology, BMC, Uppsala University, Uppsala, Sweden

**Keywords:** mast cells, influenza, TLR3, ST2, mouse model

## Abstract

Mast cells release disease-causing mediators and accumulate in the lung of asthmatics. The most common cause of exacerbations of asthma is respiratory virus infections such as influenza. Recently, we demonstrated that influenza infection in mice triggers the recruitment of mast cell progenitors to the lung. This process starts early after infection and leads to the accumulation of mast cells. Previous studies showed that an adaptive immune response was required to trigger the recruitment of mast cell progenitors to the lung in a mouse model of allergic lung inflammation. Therefore, we set out to determine whether an adaptive immune response against the virus is needed to cause the influenza-induced recruitment of mast cell progenitors to the lung. We found that influenza-induced recruitment of mast cell progenitors to the lung was intact in *Rag2*^−/−^ mice and mice depleted of CD4^+^ cells, implicating the involvement of innate immune signals in this process. Seven weeks after the primary infection, the influenza-exposed mice harbored more lung mast cells than unexposed mice. As innate immunity was implicated in stimulating the recruitment process, several compounds known to trigger innate immune responses were administrated intranasally to test their ability to cause an increase in lung mast cell progenitors. Poly I:C, a synthetic analog of viral dsRNA, induced a TLR3-dependent increase in lung mast cell progenitors. In addition, IL-33 induced an ST2-dependent increase in lung mast cell progenitors. In contrast, the influenza-induced recruitment of mast cell progenitors to the lung occurred independently of either TLR3 or ST2, as demonstrated using *Tlr3*^−/−^ or *Il1rl1*^−/−^ mice. Furthermore, neutralization of IL-33 in *Tlr3*^−/−^ mice could not abrogate the influenza-induced influx of mast cell progenitors to the lung. These results suggest that other innate receptor(s) contribute to mount the influx of mast cell progenitors to the lung upon influenza infection. Our study establishes that mast cell progenitors can be rapidly recruited to the lung by innate immune signals. This indicates that during life various innate stimuli of the respiratory tract trigger increases in the mast cell population within the lung. The expanded mast cell population may contribute to the exacerbations of symptoms which occurs when asthmatics are exposed to respiratory infections.

## Introduction

Mast cells are rare immune cells which function as sensors in innate immunity and play a pivotal role in IgE-mediated allergic reactions. In mice, mast cells have their roots in the bone marrow where committed mast cell progenitors (MCp) develop (1–3). At steady state, low frequencies of committed MCp, in the order of around 30–70 MCp per 10^6^ isolated mononuclear cells, migrate from the blood into peripheral tissues where they may mature into granulated mast cells (4). We recently discovered a similar MCp population in peripheral human blood (5). The human MCp were equally rare as the mouse counterparts, and had a progenitor-like morphology with no or a few granules.

In asthmatic patients, mast cells release pathogenic mediators and are also found in increased numbers at particular sites of the lung such as in the smooth muscle and the airway epithelium (6–8). In a mouse model of experimental asthma, MCp were recruited into the lung and led to the appearance of mast cells (9–11), suggesting that the increase in lung mast cells in asthmatics is due to the recruitment and maturation of MCp. The development of an adaptive immune response, i.e., CD11c^+^ cells and CD4^+^ T cells, were essential for the recruitment of MCp to the lung to be induced in this model of experimental asthma (11, 12). Lately, we demonstrated that MCp are also recruited to the lung in a mouse model of H1N1 influenza A infection, leading to accumulation of mast cells in the central upper airways close to inflamed bronchioles and blood vessels (13).

In mice, influenza infection is sensed by toll-like receptor 3 (TLR3), TLR7, retinoid acid-inducible gene I (RIG-I), melanoma differentiation associated protein 5 (MDA-5), and the NOD-like receptor family member NOD-, LRR- and pyrin domain-containing 3 (NLRP3) (14, 15). Activation of these receptors leads to release of type I interferons, pro-inflammatory cytokines, and eicosanoids, which orchestrate the following innate and adaptive immune responses. Influenza infection also induces the production of endogenous molecules that act as strong activators of the immune response. One such molecule is IL-33, which is released from damaged airway epithelial cells and alveolar macrophages early upon influenza infection (16, 17). IL-33 signals through the ST2 receptor expressed by e.g., mast cells and innate lymphoid cells type 2 (18), as well as epithelial cells (19). The IL-33/ST2 axis is of major importance for the development of allergic asthma, and the pathway needs to be disrupted to resolve allergic inflammation and airway hyperresponsiveness (20, 21). This pathway is also implicated in the initiation and progression of pulmonary fibrosis (22). Using *Rag2*-deficient mice, the significance of innate immune cells being induced by the IL-33/ST2 pathway has been shown by intranasal IL-33 injections (23) and influenza infection (17). Furthermore, IL-33 released due to allergen exposure leads to suppressed innate anti-viral immunity (24), demonstrating the central role of this pathway in the interplay between asthma and respiratory infections.

In the present study, we investigated whether activation of innate immunity is sufficient for the influenza-induced recruitment of MCp to the lung and whether the development of adaptive immunity is required. We used depleting antibodies and *Rag2*^−/−^ mice, and found that adaptive immune responses were dispensable for the influenza-induced recruitment of MCp to the lung. Moreover, we demonstrate that i.n. treatment of mice with Poly I:C or IL-33 alone was sufficient to enhance the number of lung MCp via activation of TLR3 and ST2, respectively. However, absence of either TLR3 or ST2 alone, or blocking IL-33 in *Tlr3*^−/−^ mice did not influence the massive influenza-induced recruitment of MCp to the lung, implicating that other pathways are involved in this process. Nevertheless, our findings uncover that innate signals alone are sufficient to trigger the recruitment of MCp to the lung.

## Materials and methods

### Animals

At least 6 weeks old, age- and sex-matched mice on the BALB/c background were used in the experiments. Wild type BALB/c mice were originally obtained from Bommice (Ry, Denmark), and *Rag2*^−/−^ mice on BALB/c background (129S6/SvEvTac-Rag2^tm1Fwa^) were obtained from Taconic Biosciences (Hudson, NY). *Tlr3*^−/−^ mice on BALB/c background were originally purchased from Oriental Bio Service Inc. (Kyoto, Japan), and the *Il1rl1*(*St2*) ^−/−^ mice on BALB/c background were provided by Dr. Andrew McKenzie (25). The *Tlr3*^−/−^ and *Il1rl1*^−/−^ mice were crossed with the in-house BALB/c mice and the resulting heterozygous offsprings used as breeding pairs to produce homozygotes for the experiments. All mice were bred and housed in the animal facility at the National Veterinary Institute (SVA), Uppsala, Sweden. This study was carried out in accordance with the recommendations of Jordbruksverket. The protocols were approved by Uppsala Djurförsöksetiska nämnd (C202/11; 5.8.18-05248/2018) and Stockholms Djurförsöksetiska nämnd (N14/16).

### Influenza infection, depletion of CD4^+^ cells, i.n. injections, and neutralization of IL-33

For influenza infection, mice were anesthetized by inhaled isoflurane (3%) followed by i.n. inoculation of 4 × 10^4^ TCID_50_ influenza A (Puerto Rico/8/34) or equal volume of PBS. Weight was monitored during the course of the experiment and mice that lost more than 15% of their initial body weight prior to the planned termination date or did not lose weight at all were euthanized and excluded from the study. To deplete CD4^+^ cells, lightly anesthetized mice received an i.p. injection of 200 μg anti-CD4 (rat IgG2b anti-mouse CD4) monoclonal antibodies on days −2, 0, 2, 4, and 6. The anti-mouse CD4 antibodies were prepared in-house from the hybridoma cell line GK 1.5. In parallel, another group of PR8-infected mice received isotype-matched antibodies [rat IgG2b anti-ovalbumin (OVA)] as controls. The anti-OVA antibodies were prepared in-house from the hybridoma cell line O4B86. The supernatants from the hybridomas were purified on a Protein G Sepharose column (GE Healthcare Life Sciences, Uppsala, Sweden) according to the manufacturer's instruction. For passive serum transfer experiments, mice received 30 μl pooled serum (15 μl in each nostril) on days −1 and 0 from mice that were either influenza-infected or PBS-injected 48 and 8 days earlier. Two hours after serum instillation on day 0, the mice were infected with PR8. For Poly I:C and R848 treatments, lightly anesthetized mice received a daily i.n. injection of 50 μg high molecular weight Poly I:C VacciGrade™ or 50 or 200 μg R848 VacciGrade™ (Invivogen, San Diego, CA, USA) or sterile endotoxin-free physiological water as vehicle for 4 consecutive days. One day after the last injection, mice were euthanized and the lungs were collected for cell isolation. For IL-33 treatments, a daily i.n. dose of 0.5 μg recombinant mouse IL-33 (Peprotech, Rocky Hill, NJ, USA) or bovine serum albumin (BSA) 0.1% in PBS as vehicle was administered to the mice under light anesthesia for 3 consecutive days. One day after last injection, mice were euthanized and lungs were collected for cell isolation. To block IL-33/ST2 axis, influenza-infected *Tlr3*^−/−^ mice were given an i.p. injection of 3.6 μg anti-IL-33 (goat IgG anti-mouse IL-33; #AF3626) polyclonal antibodies 2 h before infection, and day 1, 2, 3, 4, 5, and 6 post-infection. The dose was selected based on a study where anti-IL-33 antibody suppressed inflammation and airway hyperresponsivness in an OVA-model of allergic lung inflammation in mice (26). In parallel, another group of PR8-infected *Tlr3*^−/−^ mice received same dose of polyclonal normal goat IgG; #AB-108-C) as controls. Anti-IL-33 and control antibodies were from R&D Systems (Minneapolis, MN, USA).

### Isolation of lung cells

The lungs were collected after removing the blood through injection of 10 ml PBS into the right ventricle of the heart. Dissociation of the lung tissue was performed using the gentleMACS Octo Dissociator and mouse lung dissociation kit (Miltenyi Biotec, Bergisch Gladbach, Germany) according to manufacturer's instructions. In some experiments, the lungs were minced manually and digested with collagenase type IV (150 U/ml) (Life Technologies, Paisley, Scotland, UK) as described (27). The extracted lung cells were either enriched for mononuclear cells (MNC) using Percoll (Sigma-Aldrich, St. Louis, MO, USA) by gradient centrifugation at 400 × g with the cells resuspended in 44% Percoll and underlaid with 67% Percoll in complete RPMI (RPMI 1640, 10% heat-inactivated FCS, 100 U/ml penicillin, 100 μg/ml streptomycin, 10 μg/ml gentamicin, 0.1 mM non-essential amino acids, 2 mM L-glutamine, 10 mM HEPES, 1 mM sodium pyruvate, and 20 μM 2-mercaptoethanol; all from Sigma-Aldrich) or directly centrifuged in 44% Percoll in complete RPMI and subsequently treated with erythrocyte lysis buffer (150 mM NH_4_Cl, 10 mM KHCO_3_, 1 mM Na_2_EDTA, pH 7.3) to remove the tissue residues and red blood cells. Viable cells were counted on a hemacytometer using trypan blue exclusion.

### Quantification of lung cell populations

The lung MCp were quantified by flow cytometry except for **Figures 3A**, and Supplementary Figure 3A in which the lung MCp were quantified using a limiting dilution and clonal expansion assay (27). For flow cytometry-based quantification of lung mast cell populations, extracted lung MNC or lung cells were incubated with Alexa Flour 700-labeled anti-CD45 (30-F11), PE-Cy7-labeled anti-c-kit (2B8), PE-Cy5-labeled anti-CD3 (17A2), anti-CD4 (GK1.5), anti-CD8b (eBioH35-17.2), anti-CD11b (M1/70), anti-CD19 (ebio1D3), anti-Gr-1 (RB6-8C5), anti-B220 (RA3-6B2), anti-TER-119 (TER-119), PE-labeled anti-FcεRIα (MAR-1), Brilliant Violet 605-labeled anti-CD16/32 (2.4G2), FITC-labeled anti-integrin β7 (FIB504), and Brilliant Violet 421-labeled anti-ST2 (DIH9) or biotinylated anti-ST2 (DJ8) followed by incubation with streptavidin-APC. To assess the efficiency of CD4^+^ cell depletion, all extracted lung cells were pre-incubated with Fc-block (2.4G2) followed by incubation with PE-Cy7-labeled anti-CD45 (30-F11), Brilliant Violet 605-labeled anti-CD3 (17A2), Brilliant Violet 421-labeled anti-CD4 (RM4-4) and PE-Cy5-labeled anti-CD8b (eBioH35-17.2) monoclonal antibodies. Dead cells were excluded by staining the lung cells with Calcein AM Viability Dye (eBioscience, Hatfield, UK). For quantification of neutrophils, the lung cells were pre-incubated with Fc-block (2.4G2) followed by incubation with PE-Cy7-labeled anti-CD45 (30-F11) and PE-Cy5-labeled anti-Gr-1 (RB6-8C5) monoclonal antibodies. In all experiments, FMO controls with the appropriate isotype antibody were used. The antibodies were from BD Biosciences (Franklin Lakes, NJ, USA), eBioscience (Hatfield, UK), BioLegend (San Diego, CA, USA) or MD Bioproducts (Zürich, Switzerland). Stained cells were analyzed on a LSRII, LSR Fortessa (BD Biosciences) or CytoFLEX S flow cytometer (Beckman Coulter, Inc., Brea, CA, USA) and data were analyzed with FlowJo software (TreeStar Inc., Ashland, OR, USA).

### Statistical analysis

Statistical differences between groups were assessed using either unpaired, two-tailed Student's *t*-test or one-way ANOVA with Tukey's multiple comparisons test. The *p* < 0.05 was considered significant (^*^p < 0.05, ^**^p < 0.01, ^***^p < 0.001). The graphs were prepared using GraphPad Prism 5.0c (GraphPad software Inc., San Diego, CA).

## Results

### Influenza-induced recruitment of mast cell progenitors to the lung is intact in mice depleted of CD4^+^ cells and in *Rag2*^−/−^ mice

**Figure 1:**
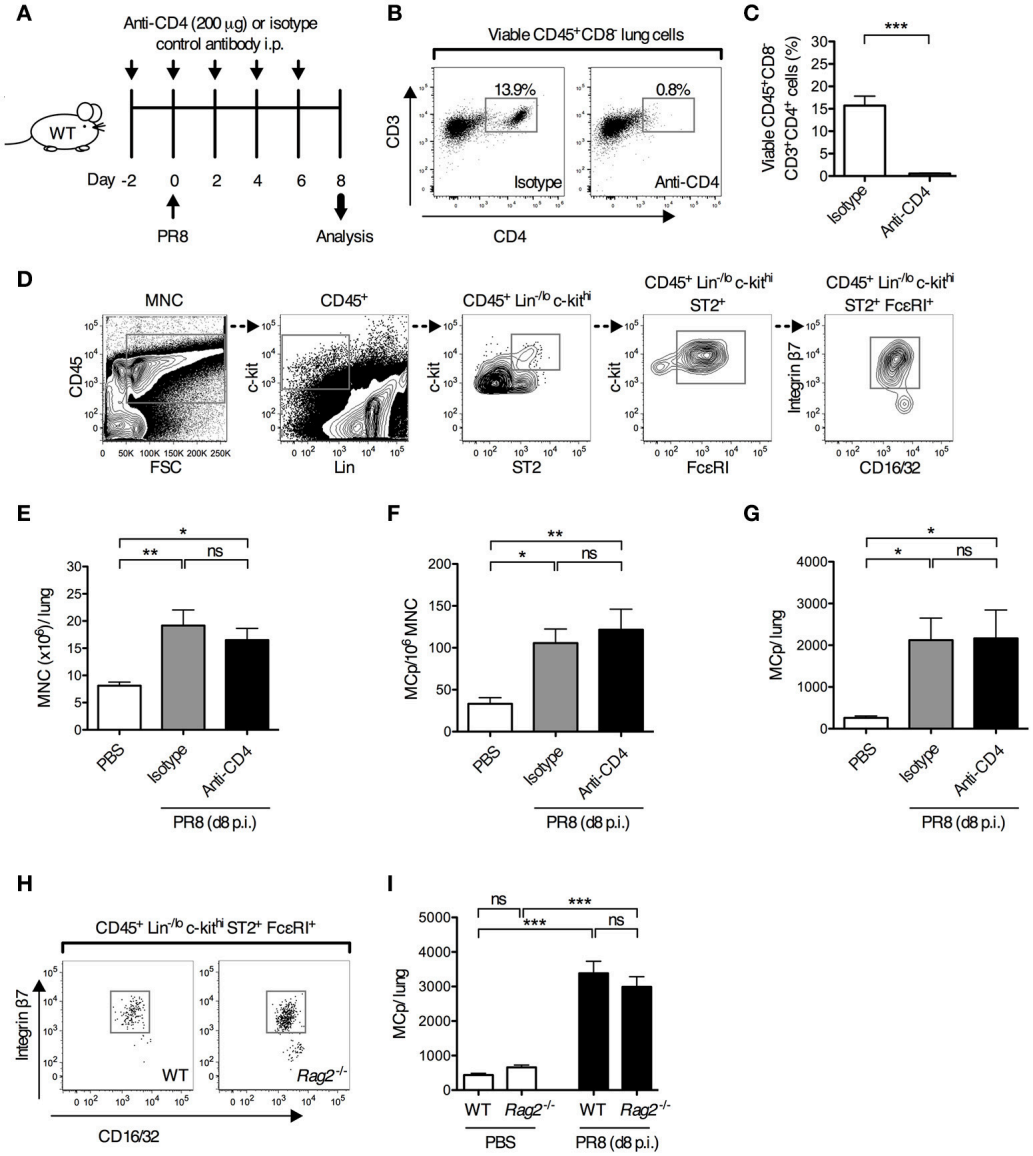
Adaptive immune responses are dispensable for influenza-induced recruitment of MCp to the lung. **(A–G)** Wild type mice were given anti-CD4 or isotype-matched antibodies (IgG2b) i.p. on days−2, 0, 2, 4 and 6 days post-influenza infection. Additional controls were given PBS i.n. day 0. **(B)** Representative dot plots showing the effect of anti-CD4 or isotype antibody injections on the frequency of lung CD4^+^ T cells (viable CD45^+^CD8^−^CD3^+^CD4^+^ lung cells). **(C)** The frequency of CD4^+^ T cells (CD45^+^CD8^−^CD3^+^CD4^+^ cells/lung lymphocytes). The data was analyzed by unpaired, two-tailed Student's *t*-test. **(D)** Representative gating strategy is shown for an influenza-infected wild type mouse. MCp were identified as CD45^+^ Lin^−/lo^ c-kit^hi^ ST2^+^ FcεRI^+^ CD16/32^int^ integrin β7^hi^ cells. **(E)** The yield of lung MNC per mouse. **(F)** The frequency of lung MCp (MCp/10^6^ MNC). **(G)** The total number of MCp per mouse lung. The results in **(C)**, and **(E–G)** are pooled from two independent experiments (*n* = 6–7). **(H,I)** Wild type (WT) and *Rag2*^−/−^ mice were infected with influenza virus or instilled with PBS. **(H)** Representative dot plots showing the frequency of lung MCp in WT and *Rag2*^−/−^ mice on day 8 post-infection. **(I)** The total number of MCp per mouse lung. The data are pooled from three independent experiments (*n* = 8–9). All data are expressed as mean ± SEM. ns = no significant difference. The data in **(E–G**,**I)** were analyzed by one-way ANOVA with Tukey's multiple comparisons test.

We have previously shown that MCp are recruited to the lung as early as 4 days post-infection with the H1N1 strain A/Puerto Rico/8/34 of influenza virus (13). As adaptive immune responses are required to promote the recruitment of MCp to the lung in a mouse model of allergic airway inflammation (11, 12), this led us to test whether development of adaptive immune responses is needed to promote the recruitment of MCp to the lung upon influenza infection. To test this, mice were given depleting anti-CD4 or isotype control antibodies days −2, 0, 2, 4, and 6 before and after influenza infection (Figure 1A). The vast majority of the CD4^+^ lung cells were CD3^+^ CD4^+^ T helper cells (Figure 1B). The anti-CD4 treatment efficiently depleted the CD4^+^ cells in the lung (Supplementary Figure 1A and Figures 1B,C). Day 8 post-infection, the mice were euthanized and lung MNC were quantified for their content of MCp, defined as CD45^+^ Lin^−/lo^ c-kit^hi^ ST2^+^ FcεRI^+^ CD16/32^int^ integrin β7^hi^ cells (Figure 1D). Influenza-infected mice given anti-CD4 antibodies had a similar yield of lung MNC as influenza-infected mice given isotype control antibodies (Figure 1E). Influenza-infected mice given anti-CD4 or isotype antibodies also had a similar frequency and total number of lung MCp (Figures 1F,G), suggesting that lung CD4^+^ T cells are dispensable for influenza-induced recruitment of MCp to the lung. To address whether adaptive immunity as a whole is dispensable for this effect, wild type and *Rag2*^−/−^ mice, which lack mature T and B cells, were infected in parallel with influenza virus. At day 8 post-infection, the frequency of lung MCp was higher and the yield of lung MNC was lower in *Rag2*^−/−^ mice than in their wild type counterparts, likely due to the lack of lymphocytes (Figure 1H, Supplementary Figures 1B,C). Nevertheless, the total number of lung MCp was comparable between influenza-infected *Rag2*^−/−^ and the control mice (Figure 1I), showing that adaptive immune responses are not required for the recruitment of MCp to the lung upon influenza infection and implying that innate immunity is sufficient to induce the recruitment-response of MCp upon influenza infection.

### Adaptive immune responses suppress the recruitment of mast cell progenitors to the lung upon a secondary infection with influenza

**Figure 2:**
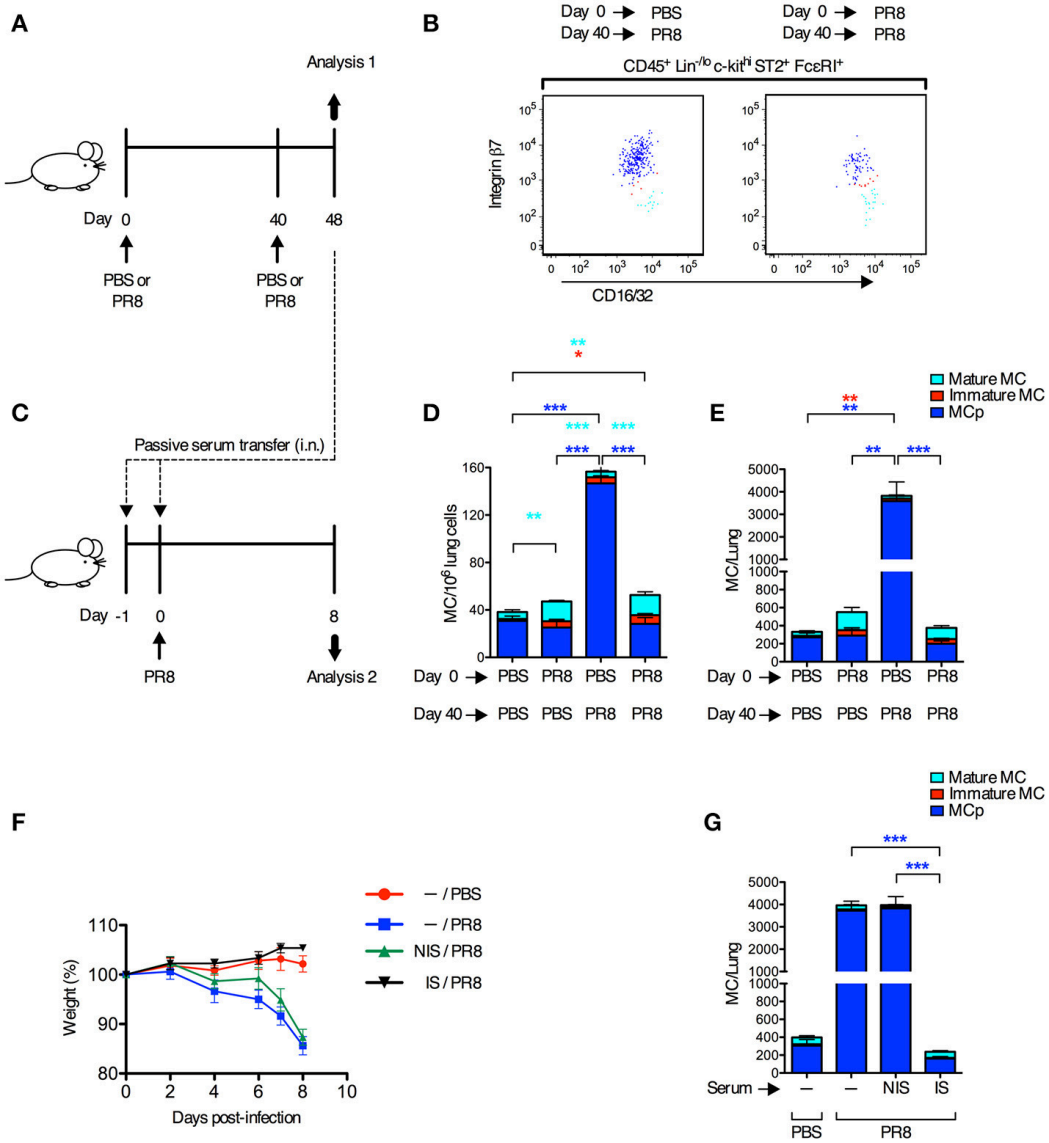
Adaptive immune responses suppress the recruitment of MCp to the lung upon a secondary infection with influenza. **(A,B,D,E)** Forty days after PR8 influenza infection or PBS installation, mice were infected with PR8 influenza virus or given PBS. **(B)** Representative dot plots of the three lung subpopulations of CD45^+^ Lin^−/lo^ c-kit^hi^ ST2^+^ FcεRI^+^ CD16/32^int^ mast cells (MC), which were distinguished based on cell surface expression level of integrin β7, as lung MCp (integrin β7^hi^; dark blue), immature MC (integrin β7^int^; red), and mature MC (integrin β7^−/lo^; light blue). **(D)** The frequency (MC/10^6^ lung cells) and total number **(E)** of MC subpopulations per mouse. The results in **(D,E)** are pooled from two independent experiments (*n* = 5–9). Mean ± SEM, one-way ANOVA with Tukey's multiple comparisons test. **(C,F,G)** Naïve mice received pooled serum from influenza-infected (immune serum; IS) or PBS-injected mice (non-immune serum; NIS) i.n. on days −1 and 0 before PR8 infection. Controls were given only PBS or PR8 on day 0. **(F)** The weight per mouse relative to the weight at day 0 (weight %). **(G)** The total number of MCp per mouse lung. The results in **(F,G)** are pooled data from two independent experiments (*n* = 6–9). Mean ± SEM, one-way ANOVA with Tukey's multiple comparisons test.

Next, we tested whether innate immune responses could stimulate the recruitment of MCp to the lung in the presence of a fully developed adaptive immune response toward the same virus. Mice were infected with influenza virus or received PBS, 40 days after a primary influenza infection or PBS instillation (Figure 2A). On day 48, lungs were analyzed for the frequency and total number of the three different mast cell subpopulations that were expected to be present at this late time point after the primary infection, i.e., mature mast cells (integrin β7^−/lo^), immature mast cells (integrin β7^int^) and MCp (integrin β7^hi^) as defined (13) (Figure 2B). As expected, mice receiving PBS day 0 and influenza virus day 40, demonstrated a 5- and 13-fold increase in the frequency and total number of lung MCp respectively, in comparison to mice given PBS at both occasions (Figures 2D,E). There were no differences in the frequency and total number of lung MCp (dark blue bars) between mice receiving influenza virus day 0 and day 40, and mice receiving PBS day 0 and day 40, or influenza virus day 0 and PBS day 40 (Figures 2D,E). This illustrates that the frequency and total number of lung MCp has returned to basal levels 48 days after the primary infection and that development of adaptive immune responses after the primary influenza infection protects the mice from a new wave of influenza-induced recruitment of MCp to the lung during the secondary infection. Indeed, the mice infected with influenza virus day 0 and re-infected day 40 were also protected from influenza-induced weight loss and had no significant increase in the number of lung cells (Supplementary Figures 2A,B). Nevertheless, the groups of mice that received the primary influenza infection (PR8 day 0) had a higher frequency and/or showed a tendency to have a higher frequency and total number of both immature (red bars) and mature mast cells (turquoise bars) at day 48 than mice that only received PBS (Figures 2D,E). These data illustrate that almost 7 weeks post-infection with influenza, the mast cell burden in the lung is still higher than in control mice.

To test whether neutralizing antibodies were responsible for the protection against influenza-induced recruitment of MCp to the lung upon a secondary infection, serum from influenza-infected or PBS-injected mice were given to naïve mice 1 day and 2 h before they were infected with influenza virus (Figure 2C). Immune serum, but not serum from PBS-injected mice, efficiently protected mice from influenza-induced weight loss, and prevented an increase in lung cells and influenza-induced recruitment of MCp to the lung (Figures 2F,**G***;* Supplementary Figures 2C,D). These data demonstrate that immune serum alone can suppress the influenza infection and thereby the recruitment of MCp to the lung.

### Poly I:C and IL-33 induce an increase in lung mast cell progenitors via TLR3 and ST2, respectively

**Figure 3:**
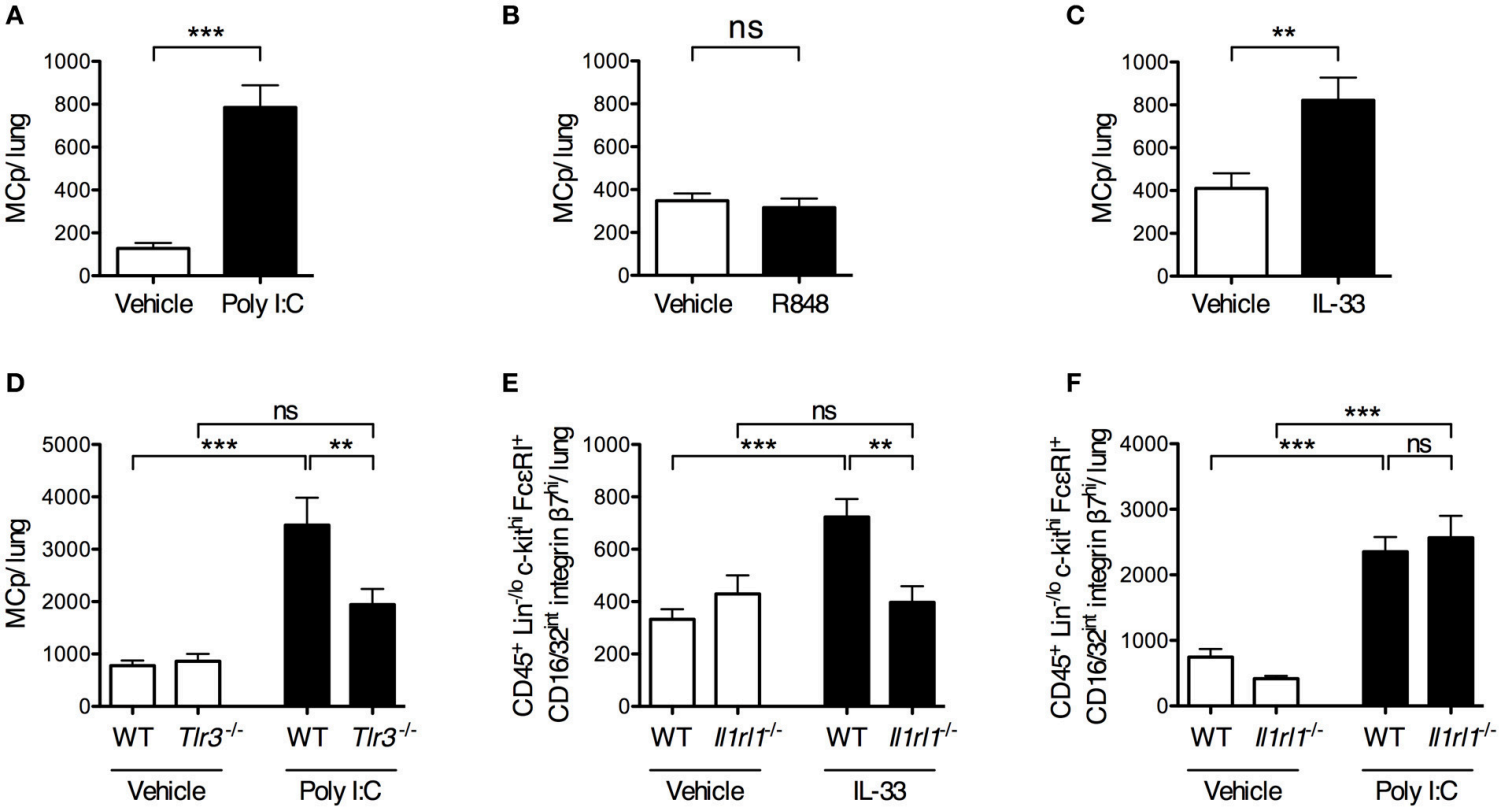
Intranasal administration of Poly I:C or IL-33 increases the number of lung MCp in a TLR3- or ST2-dependent manner. **(A,B)** The total number of MCp per mouse lung 24 h after the mice received vehicle or Poly I:C **(A)** or R848 **(B)** i.n. for 4 consecutive days. The data in **(A)** are pooled from three independent experiments (*n* = 8–12) and the data in **(B)** from two independent experiments (*n* = 9). **(C)** The total number of MCp per mouse lung 24 h after the mice received IL-33 or vehicle i.n. for three consecutive days. The data are pooled from four independent experiments (*n* = 14–16). The data in **(A–C)** were analyzed by unpaired, two-tailed Student's *t*-test. **(D)** The total number of MCp per mouse lung in wild type (WT) and *Tlr3*^−/−^ mice given Poly I:C or vehicle. The data are pooled from three independent experiments (*n* = 10). **(E)** The total number of CD45^+^ Lin^−/lo^ c-kit^hi^ FcεRI^+^ CD16/32^int^ integrin β7^hi^ cells per mouse lung gated as shown in Supplementary Figure 4. The data are pooled from three independent experiments (*n* = 8–12). **(F)** The total number of CD45^+^ Lin^−/lo^ c-kit^hi^ FcεRI^+^ CD16/32^int^ integrin β7^hi^ cells per mouse lung. The data are pooled from two independent experiments (*n* = 7–8). The data in **(D–F)** were analyzed by one-way ANOVA with Tukey's multiple comparisons test. All data are expressed as mean ± SEM. no significant difference.

To determine whether stimulation of single pattern recognition receptors is sufficient to stimulate an increased lung MCp population, mice were given i.n. injections of synthetic ligands to TLRs involved in sensing influenza virus. The TLR3 agonist Poly I:C stimulated a six-fold increase in the total number of lung MCp (Figure 3A). Also, the frequency of lung MCp were increased significantly (Supplementary Figure 3A). Next, *Tlr3*-deficient mice and their wild type littermate controls were treated in parallel with vehicle or Poly I:C. The number of lung MCp in wild type mice was elevated by Poly I:C injection, but in *Tlr3*^−/−^ mice Poly I:C failed to stimulate a significant increase in the number of MCp (Figure 3D). This suggests that Poly I:C acts via TLR3 to stimulate an increased number of lung MCp.

As TLR7 also has been implicated in sensing of influenza virus (14), the synthetic imidazoquinoline compound R848, known to activate TLR7 (28), was tested for its ability to increase the number of lung MCp. However, R848 did not increase the total number of lung MCp (Figure 3B). Instead, the frequency of lung MCp was significantly decreased although the yield of lung MNC was significantly increased upon R848 treatment, suggesting that another cell population among the MNC was stimulated to expand (Supplementary Figures 3B,C).

IL-33 is produced early after influenza infection (16), and was therefore tested for its ability to enhance the lung MCp population. IL-33 treatment resulted in a two-fold increase in the total number of lung MCp as compared to vehicle-treated mice (Figure 3C) and a 1.6-fold increase in the frequency of lung MCp (Supplementary Figure 3F). To determine whether the effect of IL-33 on the expansion of lung MCp was mediated through the ST2 receptor, *Il1rl1*^−/−^ mice and their wild type littermates received i.n. injections of IL-33 or vehicle. An alternative gating strategy was employed since ST2 could not be used as a surface marker to identify lung MCp in experiments that involved *Il1rl1*^−/−^ mice (Supplementary Figure 4). Thus, the frequency and total number of CD45^+^ Lin^−/lo^ c-kit^hi^ FcεRI^+^ CD16/32^int^ integrin β7^hi^ cells in *Il1rl1*^−/−^ mice and their wild type controls were compared in these experiments. In wild type mice, 92 ± 1% of these cells were expressing ST2 and were therefore defined as lung MCp (Figure 1D). As expected, the total number of CD45^+^ Lin^−/lo^ c-kit^hi^ FcεRI^+^ CD16/32^int^ integrin β7^hi^ cells increased significantly in the lung of wild type mice upon IL-33 treatment (Figure 3E). However, IL-33 failed to enhance the number of these cells in *Il1rl1*^−/−^ mice (Figure 3E).

As IL-33 expression is induced upon Poly I:C treatment (29, 30), we tested whether the enhancing effect of Poly I:C on the number of lung MCp was mediated by the IL-33/ST2-axis. However, *Il1rl1*^−/−^ mice injected with Poly I:C had a similar level of CD45^+^ Lin^−/lo^ c-kit^hi^ FcεRI^+^ CD16/32^int^ integrin β7^hi^ cells in the lung as the wild type mice treated in parallel (Figure 3F). This suggests that Poly I:C and IL-33 stimulate an increased number of lung MCp by separate mechanisms. Altogether, these results reveal that Poly I:C or IL-33 alone is sufficient to trigger an enhanced number of lung MCp by activating TLR3 or ST2, respectively.

### ST2 and TLR3 are dispensable for influenza-induced recruitment of mast cell progenitors to the lung

**Figure 4:**
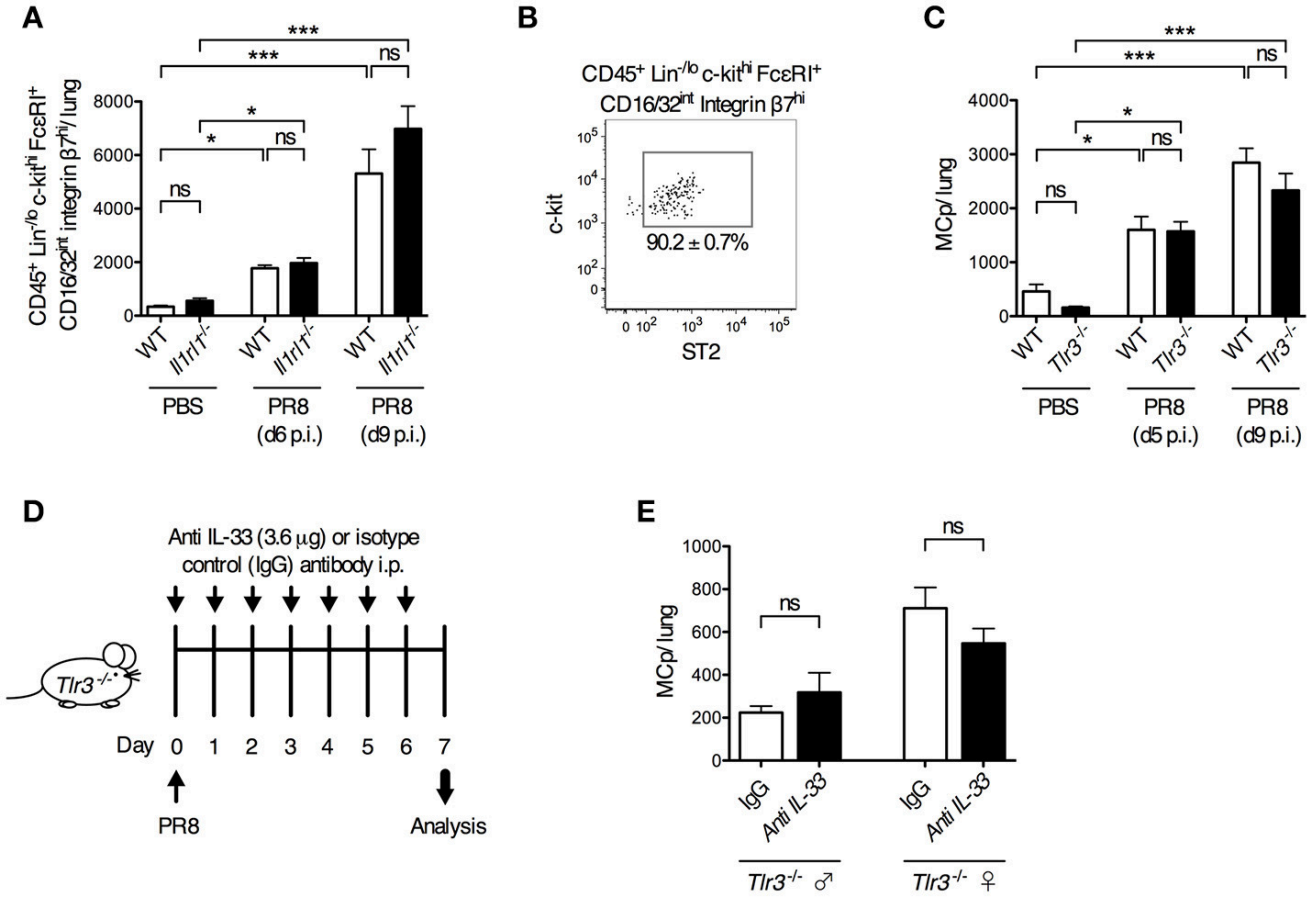
ST2- and TLR3-mediated signals are dispensable for influenza-induced recruitment of MCp to the lung*. Il1rl1*^−/−^ or *Tlr3*^−/−^ mice and their wild type (WT) littermates were infected with PR8 influenza virus or given PBS. **(A)** The total number of CD45^+^ Lin^−/lo^ c-kit^hi^ FcεRI^+^ CD16/32^int^ integrin β7^hi^ cells per mouse lung using the gating strategy shown in Supplementary Figure 4. The data are pooled from four independent experiments, three analyzed day 6 post-infection (d6 p.i.) and one analyzed d9 p.i. (*n* = 9–10 mice PBS-treated, *n* = 6–15 PR8-infected). **(B)** The majority of the CD45^+^ Lin^−/lo^ c-kit^hi^ FcεRI^+^ CD16/32^int^ integrin β7^hi^ cells from WT mice are ST2^+^. **(C)** The total number of MCp per lung. The data are pooled from four independent experiments, two analyzed d5 p.i. and two analyzed d9 p.i. (*n* = 4–10 PBS-treated, *n* = 9–13 PR8-infected). The data in **(A)** and **(C)** are expressed as mean ± SEM and analyzed by one-way ANOVA with Tukey's multiple comparisons test. ns = no significant difference. **(D–E)** *Tlr3*^−/−^ mice were given anti-IL-33 or polyclonal normal goat IgG i.p. 2 h before infection and daily from day 1 to day 6 post-influenza infection. **(E)** The total number of MCp per lung assessed on day 7 post-influenza infection. The data are pooled from two independent experiments, using *Tlr3*^−/−^ male (*n* = 4–6) and female (*n* = 5–6) mice. The data in **(E)** was analyzed by unpaired, two-tailed Student's *t*-test.

To test the role of ST2 in a live influenza virus infection, *Il1rl1*-deficient mice and their wild type littermates were infected i.n. with influenza virus or given PBS. Six and nine days post-infection, *Il1rl1*^−/−^ and wild type mice had a similar frequency and total number of CD45^+^ Lin^−/lo^ c-kit^hi^ FcεRI^+^ CD16/32^int^ integrin β7^hi^ lung cells (Supplementary Figure 5A, Figure 4A). In these experiments, 90.2 ± 0.7% of the gated CD45^+^ Lin^−/lo^ c-kit^hi^ FcεRI^+^ CD16/32^int^ integrin β7^hi^ lung cells in wild type mice were ST2^+^ (Figure 4B). Hence, the ST2/IL-33 pathway is dispensable for influenza-induced MCp recruitment to the lung.

Next, we investigated whether TLR3 is involved in the influenza-induced recruitment of MCp to the lung. However, influenza infection stimulated a similar increase in the frequency and total number of lung MCp in both *Tlr3*^−/−^ and wild type mice (Supplementary Figure 5B, Figure 4C). This demonstrates that the influenza-induced recruitment of MCp to the lung occurs independently of TLR3. To test whether the IL-33/ST2 and TLR3 pathways can function redundantly to recruit MCp to the lung upon influenza infection, IL-33 neutralizing antibodies or control goat IgG were administrated before and after influenza infection in *Tlr3*^−/−^ mice (Figure 4D). Nevertheless, the neutralizing anti-IL-33 antibodies could not reduce the influx of MCp to the lung in the *Tlr3*^−/−^ mice (Figure 4E). Altogether, our data indicate that TLR3- and ST2-mediated signals are dispensable for influenza-induced recruitment of MCp to the lung, and that other receptors are involved in this process.

## Discussion

The prominent role of respiratory viruses in causing exacerbations of asthma symptoms prompted us to study mast cells in the context of influenza infection in mice. Here, we uncovered that innate signals alone are sufficient to trigger the recruitment of MCp to the lung in influenza infection. This finding was demonstrated by showing that the recruitment of MCp to the lung, occurring early after influenza infection (13), is unperturbed in mice lacking CD4^+^ T cells or all types of T and B cells (*Rag2*^−/−^ mice). Although adaptive immunity was dispensable for the recruitment of MCp to the lung, mice which were re-infected after 40 days were protected from another influx of MCp to the lung. The protective effect could be transferred by immune serum, suggesting that specific antibodies, and B cells played a crucial role in this process. We conclude that the recruitment of MCp to the lung is truly an innate response during primary influenza infection. The mechanism behind the protective effect of the adaptive response during a secondary infection is likely related to specific antibodies that efficiently neutralize the influenza virus and prevent the stimulation of receptors involved in sensing of influenza infection.

The fact that innate immunity plays a key role for the influenza-induced recruitment of MCp to the lung, implies that the MCp are more easily recruited to the lung than the pioneering studies of OVA-induced recruitment of MCp to the lung in model of allergic airway inflammation suggested. In the OVA model, lymphocytes become activated and expand during the sensitization phase when OVA is given intraperitoneally. Upon OVA aerosol challenge, cytokine-producing CD4^+^ T effector cells are recruited to the lung. These cells are required for the OVA-induced upregulation of molecules such as VCAM-1, which is crucial for the recruitment of MCp to the lung (9, 12). Hence, the recruitment of MCp to the lung can be stimulated by various inflammatory processes in the lung, either by CD4^+^ T lymphocytes during allergic inflammation or by innate immune reactions occurring during primary influenza infection, which also induce VCAM-1 expression on the lung endothelium (13). As shown in the present study, lymphocytes, i.e., antibody-producing B cells can also prevent the MCp recruitment process during a secondary infection.

The lack of importance of adaptive immunity for the recruitment of MCp to the lung upon influenza infection prompted us to investigate receptors involved in sensing of influenza virus. We identified TLR3 and ST2 as receptors which could be stimulated through i.n. injections of Poly I:C and IL-33, respectively, to increase the lung MCp population by 2- to 6-fold. A significant role of TLR3- and ST2-signaling in the immune response against influenza infection was demonstrated before (16, 17, 31). However, in the present study both *Tlr3-* and *Il1rl1*-deficient mice had an intact recruitment of MCp to the lung in response to the influenza infection and blocking the IL-33/ST2 pathway in *Tlr3*^−/−^ mice could not either abrogate the influx. Although these results may seem counter-intuitive, the activation of the TLR3- or the IL-33/ST2 pathways alone triggered only a few-fold increase in lung MCp, which is by far not as impressive as the massive recruitment of MCp to the lung induced by influenza infection. Nevertheless, these findings suggest that other receptors involved in sensing influenza infection are critical for or are contributing to the induction of the recruitment of MCp to the lung. For example, TLR7 is implicated as a endosomal receptor involved in sensing influenza virus (14). However, as TLR7 is nonessential for the production of pro-inflammatory cytokines/chemokines and development of protective immunity toward primary influenza A infection in mice (32, 33), TLR7 is likely dispensable also for influenza-induced recruitment of MCp to the lung. Moreover, we found that i.n. injection of the TLR7 ligand (R848) could not stimulate an increased number of lung MCp, although a single dose of the TLR7 ligand could recruit neutrophils (Supplementary Figures 3D,E), as shown before (34). RIG-I and MDA-5 are sensors of influenza that recognize viral replication intermediates in the cytosol. Therefore, they cannot be targeted by i.n. injection of synthetic RNA species. *Rig-I* (*Ddx58*)^−/−^ mice show defects in antigen presentation and priming of T cell responses upon infection with PR8 influenza virus (35). Unfortunately, deletion of *Rig-I* is embryonically lethal in BALB/c and C57BL/6 mice (36). A possible way to get viable *Rig-I*^−/−^ mice is to cross *Rig-I*^+/−^ mice with ICR (Institute of Cancer Research) mice, and then to intercross them to generate F2 mice, which was used in (35). Therefore, RIG-I is a likely candidate to be involved in influenza-induced recruitment of MCp to the lung. While RIG-I has been identified as the major sensor of viral replication, MDA-5 is only implicated as a minor contributor during influenza A infection (15). Finally, influenza infection triggers NLRP3 activation, which lead to formation of large multimeric complexes called inflammasomes that cause release of IL-1/IL-18 and pyroptosis (37). However, since this receptor requires a second sensing receptor to be activated, NLRP3 cannot solely be required for the recruitment of MCp to the lung, but may also be involved. We speculate that most likely several innate receptors act in concert to cause the influenza-induced recruitment of MCp to the lung and that lacking a single receptor is not enough to inhibit this process.

Altogether, this study demonstrates the crucial role of innate immunity to stimulate the recruitment of MCp to the lung during primary influenza infection and indicates that multiple redundant pathways may be involved in this process. Interestingly, MCp accumulate in the lung also in response to weaker innate stimulation such as upon i.n. injections of ligands to TLR3 and ST2, although not to the same level as the live influenza infection. Nevertheless, these findings add to the previous data demonstrating that IL-33 acts via ST2 to increase mast cell surface IgE, and to trigger histamine release and systemic anaphylaxis in naïve mice (38) and that poly I:C activates mast cells via TLR3, which may result in the recruitment of CD8^+^ cells (39). The finding that the influenza-induced recruitment of MCp to the lung leads to an increased mast cell burden in the airways for an extended time (at least 7 weeks post-infection) is intriguing. This fact may explain why lung mast cells are relatively abundant in humans, which are constantly exposed to new respiratory viruses, in contrast to laboratory mice that are limitedly exposed to pathogens and harbor very few mast cells in their lungs. We also speculate that the increased lung mast cell population which is caused by respiratory virus infection may be pathological for individuals with chronic respiratory diseases such as asthma and chronic obstructive pulmonary disease, who frequently suffer from virus-induced exacerbations of their disease.

## Author contributions

BZ and JH conceived and designed the experiments; BZ and AW performed most of the experiments; SE performed one set of experiments; BZ and JH analyzed and interpreted the data; BZ and JH wrote the manuscript.

### Conflict of interest statement

The authors declare that the research was conducted in the absence of any commercial or financial relationships that could be construed as a potential conflict of interest.

## Abbreviations

MCp: mast cell progenitor(s)
MDA-5: melanoma differentiation associated protein 5
NLRP3: NOD-like receptor family member NOD-LRR- and pyrin domain-containing 3
Poly I:C: polyinosinic-polycytidylic acid
PR8: Puerto Rico 8, mouse adopted H1N1 influenza strain
RIG-I: retinoid acid-inducible gene I
TLR: toll-like receptor.
