# Supplementary material for: Innate Immunity Induces the Accumulation of Lung Mast Cells During Influenza Infection
Source: Front Immunol. 2018 Oct 4;9:2288. doi: 10.3389/fimmu.2018.02288 (PMC6180200; doi:10.3389/fimmu.2018.02288)
Supplement: Supplementary file 1 [file Data_Sheet_1.PDF]

Supplementary Material

Innate immunity induces the accumulation of lung mast cells during influenza infection

B. Zarnegar[1], A. Westin[1], S. Evangelidou [1] and J. Hallgren[1*]

[1] Department of Medical Biochemistry and Microbiology, BMC, Uppsala University,

751 23 Uppsala, Sweden.

*** Correspondence to:** Jenny Hallgren, Department of Medical Biochemistry and Microbiology,

Uppsala University, Box 582, SE-751 23 Uppsala, Sweden.

Tel.: +46 18 471 4676

Email: jenny.hallgren@imbim.uu.se

Supplementary Figures

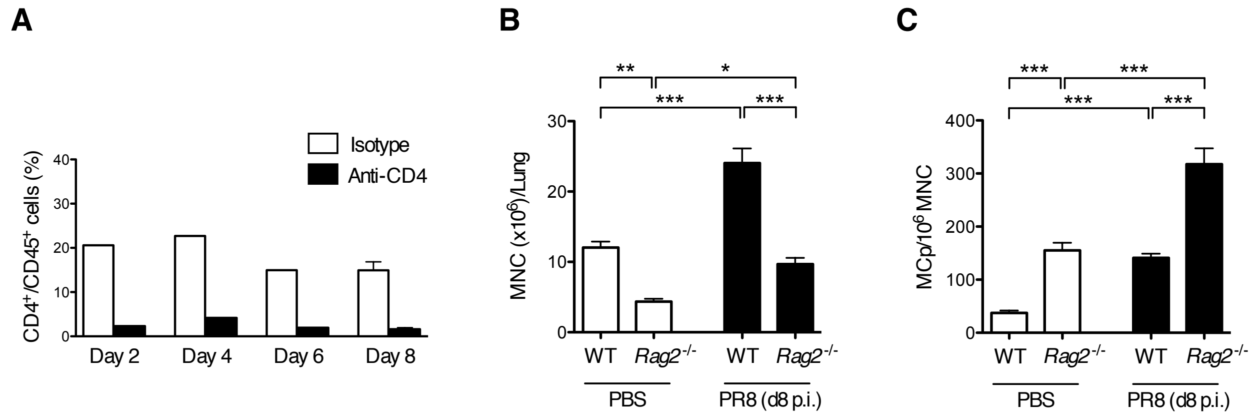

Supplementary Figure 1 (S1). Adaptive immune responses are dispensable for influenza-induced recruitment of MCp to the lung. **(A)** Wild type mice were given anti-CD4 or isotype-matched antibody (IgG2b) (200 µg) i.p. on days -2, 0, 2, 4 and 6 days post-infection with PR8 influenza virus. The mice were euthanized at day 8 post-infection and the lungs were analyzed by flow cytometry for the frequency of $CD4^+$ T cells (n = 2-3). The data is shown as mean or mean ± SEM. **(B, C)** Wild type (WT) and *Rag2*$^{-/-}$ mice were infected with PR8 influenza virus or instilled with PBS. At day 8 post-infection, mice were euthanized and the lungs were quantified for **(B)** MNC and **(C)** the frequency of lung MCp (MCp/10^6 MNC) determined by flow cytometry. The results in panels **B** and **C** are pooled from three independent experiments (n = 8-9). Mean ± SEM, one-way ANOVA with Tukey's multiple comparisons test.

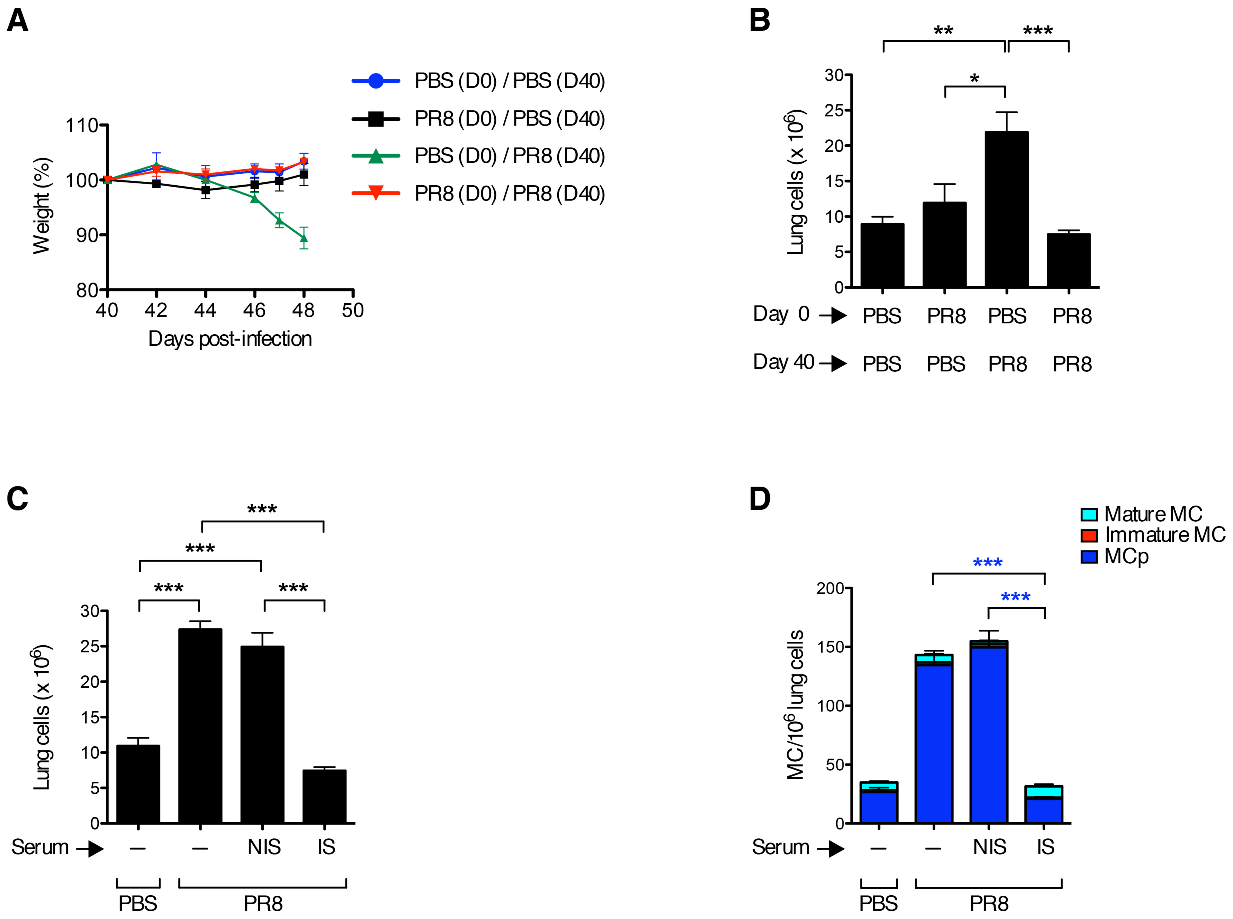

Supplementary Figure 2 (S2). Adaptive immune responses suppress the recruitment of MCp to the lung upon a secondary influenza infection. **(A, B)** Forty days after PR8 influenza infection or PBS installation, mice received either PR8 influenza virus or PBS and their lungs were analyzed on day 48. **(A)** The body weight at days 40-48 after the primary treatment normalized to each mouse's weight at day 40. **(B)** The average yield of lung cells per group of mice analyzed on day 48. The results in **A** and **B** are pooled data from two independent experiments (n = 5-9). Mean ± SEM, one-way ANOVA with Tukey's multiple comparisons test. **(C, D)** Naïve mice received pooled serum from influenza-infected (immune serum; IS) or PBS-injected mice (non-immune serum; NIS) i.n. on days -1 and 0. Two hours after serum instillation on day 0, the mice were infected with PR8. Mice given only PBS or PR8 i.n. on day 0 (without passive transfer of serum) were analyzed in parallel. On day 8 post-infection, **(C)** the number of lung cells were counted and **(D)** the frequency of mast cell (MC) subpopulations per mouse lung determined by flow cytometry. The results in **C** and **D** are pooled from two independent experiments (n = 6-9). Mean ± SEM, one-way ANOVA with Tukey's multiple comparisons test.

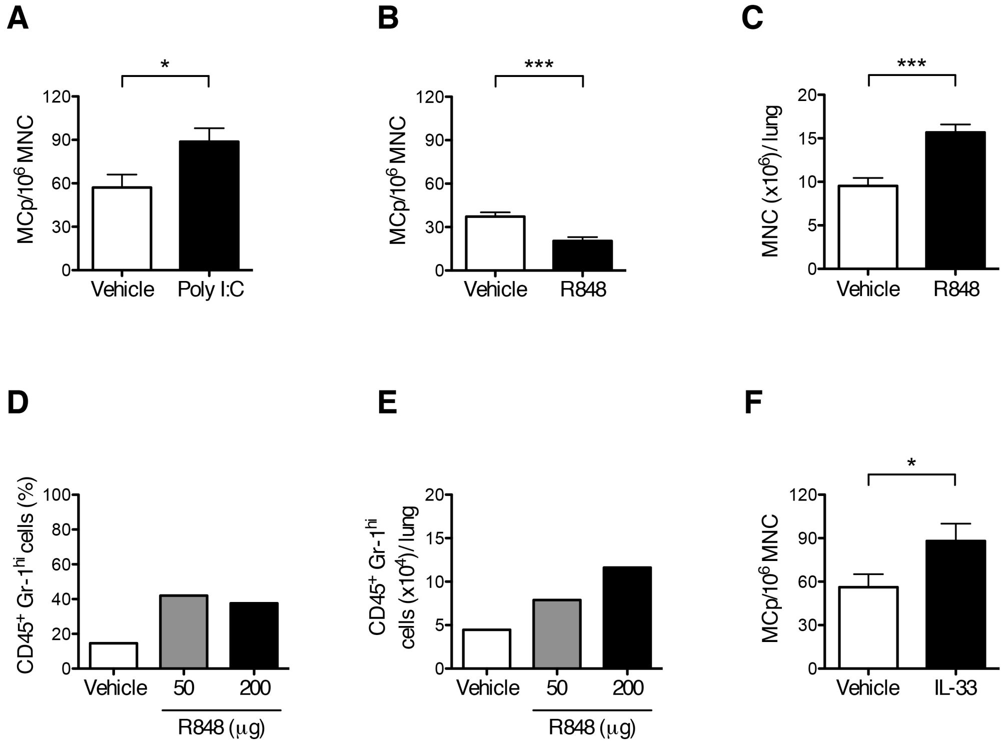

Supplementary Figure 3 (S3). Intranasal administration of Poly I:C or IL-33, but not R848, stimulates an increased frequency of lung MCp. **(A, B)** The frequency of lung MCp (MCp/10^6 MNC) per mouse 24 h after the last treatment in mice given a daily i.n. dose of 50 µg **(A)** Poly I:C or **(B)** R848 or vehicle for four consecutive days. **(C)** The total yield of lung MNC 24 h after the last treatment in mice given a daily i.n. dose of 50 µg R848 or vehicle for four consecutive days. The results in **A** are pooled from three independent experiments (n = 8-12) and the data in panels **B** and **C** are pooled from two independent experiments (n = 9). Mean ± SEM, unpaired, two-tailed Student's t-test. **(D, E)** Mice were given a single i.n. dose of 50 or 200 µg R848 or vehicle. Twenty-four hours later, the mice were euthanized and lungs were analysed for **D** the percentage and **E** the total number of $CD45^+$ Gr-1^{hi} lung cells by flow cytometry. The bars show the mean ± range from a single experiment with two mice per group. **(F)** The average frequency of lung MCp (MCp/10^6 MNC) per mouse in mice given a daily i.n. dose of 0.5 µg IL-33 or vehicle for three consecutive days. The mice were euthanized 24 h after the last IL-33 treatment for analysis. The graphs are pooled data from four independent experiments (n = 14-16). Mean ± SEM, unpaired, two-tailed Student's t-test.

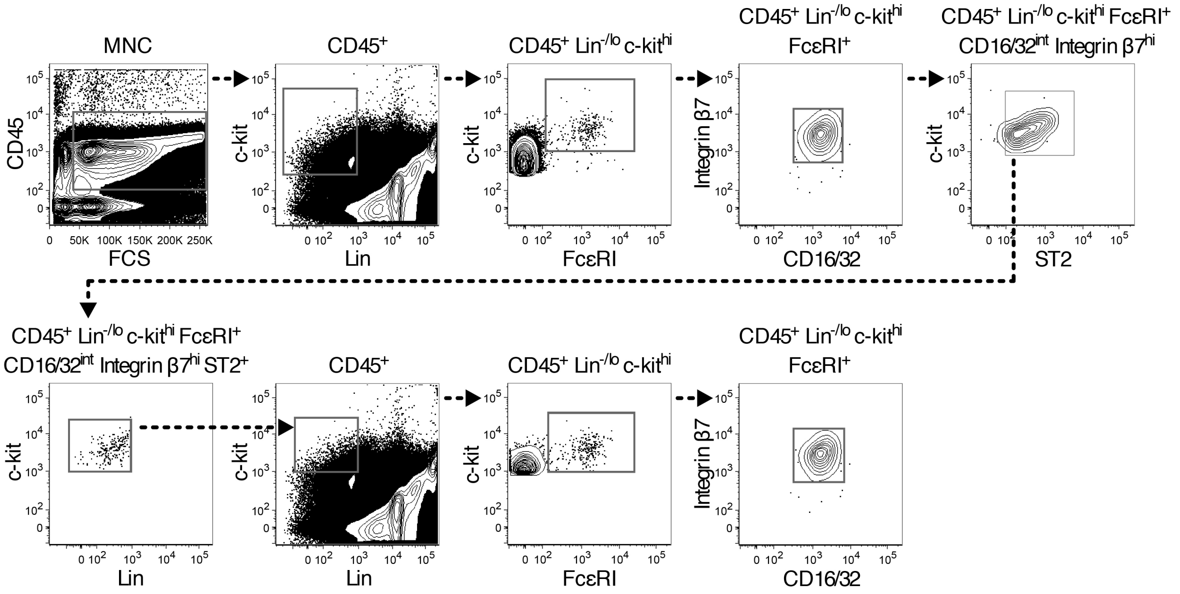

Supplementary Figure 4 (S4). Gating strategy used for quantification of CD45$^+$ Lin$^{-/lo}$ c-kit^{hi} FcεRI$^+$ CD16/32^{int} integrin β7^{hi} cells in the experiments comparing *Il1rl1*$^{-/-}$ and wild type mice. The CD45$^+$ Lin$^{-/lo}$ c-kit^{hi} FcεRI$^+$ CD16/32^{int} integrin β7^{hi} cells were gated in wild type mice (upper panel). Next, the ST2$^+$ CD45$^+$ Lin$^{-/lo}$ c-kit^{hi} FcεRI$^+$ CD16/32^{int} integrin β7^{hi} cells were back-gated to find where the MCp were located on the c-kit/Lin axis among the CD45$^+$ Lin$^{-/lo}$ c-kit^{hi} FcεRI$^+$ CD16/32^{int} integrin β7^{hi} cells (lower panel). From that, a more limited gate was chosen as a starting gate to gate the CD45$^+$ Lin$^{-/lo}$ c-kit^{hi} FcεRI$^+$ CD16/32^{int} integrin β7^{hi} cells (lower panel). The sample shown is a wild type mouse treated with IL-33.

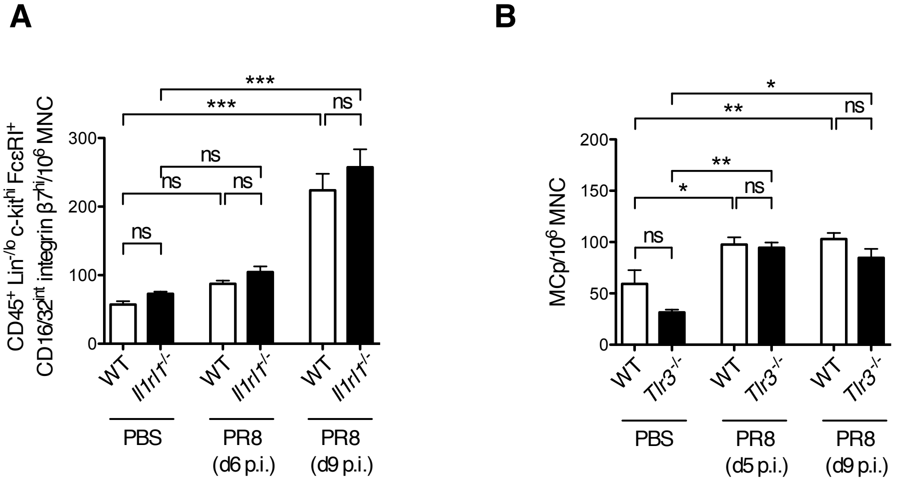

Supplementary Figure 5 (S5). ST2- and TLR3-mediated signals are dispensable for influenza-induced recruitment of MCp to the lung. *Il1rl1*$^{-/-}$ and/or *Tlr3*$^{-/-}$ mice and their wild type (WT) littermates were infected with PR8 influenza virus or given PBS. **(A)** The frequency of CD45^{+} Lin$^{-/lo}$ c-kit^{hi} FcεRI^{+} CD16/32^{int} integrin β7^{hi} cells per mouse lung using the gating strategy shown in Supplementary Figure 4. The data are pooled from four independent experiments, three analyzed d6 p.i. and one analyzed d9 p.i. (n = 9-10 PBS-treated, n= 6-15 PR8-infected). **(B)** The frequency of lung MCp. The data are pooled from four independent experiments, two analyzed day 5 post-infection (d5 p.i.) and two analyzed d9 p.i. (n = 4-10 PBS-treated, n = 9-13 PR8-infected). Mean ± SEM, one-way ANOVA with Tukey's multiple comparisons test. ns = not significantly different.
